# Cardiorespiratory consequences of attenuated fentanyl and augmented rocuronium dosing during protocolised prehospital emergency anaesthesia at a regional air ambulance service: a retrospective study

**DOI:** 10.1186/s13049-024-01183-4

**Published:** 2024-02-12

**Authors:** Sarah Morton, Zoey Spurgeon, Charlotte Ashworth, James Samouelle, Peter B Sherren

**Affiliations:** 1 Essex & Herts Air Ambulance Trust, Essex, UK; 2 Imperial College London, London, UK; 3 Guy’s and St Thomas’ NHS Foundation Trust, London, UK

**Keywords:** Pre-hospital anaesthesia, Trauma, Hypoxia, Hypotension, Drug regimens

## Abstract

**Background:**

Pre-Hospital Emergency Anaesthesia (PHEA) has undergone significant developments since its inception. However, optimal drug dosing remains a challenge for both medical and trauma patients. Many prehospital teams have adopted a drug regimen of 3 mcg/kg fentanyl, 2 mg/kg ketamine and 1 mg/kg rocuronium (‘3:2:1’). At Essex and Herts Air Ambulance Trust (EHAAT) a new standard dosing regimen was introduced in August 2021: 1 mcg/kg fentanyl, 2 mg/kg ketamine and 2 mg/kg rocuronium (up to a maximum dose of 150 mg) (‘1:2:2’). The aim of this study was to evaluate the cardiorespiratory consequences of a new attenuated fentanyl and augmented rocuronium dosing regimen.

**Methods:**

A retrospective study was conducted at EHAAT as a service evaluation. Anonymized records were reviewed from an electronic database to compare the original (‘3:2:1’) drug dosing regimen (December 2019-July 2021) and the new (‘1:2:2’) dosing regimen (September 2021-May 2023). The primary outcome was the incidence of absolute hypotension within ten minutes of induction. Secondary outcomes included immediate hypertension, immediate hypoxia and first pass success (FPS) rates.

**Results:**

Following exclusions (*n* = 121), 720 PHEA cases were analysed (360 new vs. 360 original, no statistically significant difference in demographics). There was no difference in the rate of absolute hypotension (24.4% ‘1:2:2’ v 23.8% ‘3:2:1’, *p* = 0.93). In trauma patients, there was an increased first pass success (FPS) rate with the new regimen (95.1% v 86.5%, *p* = 0.01) and a reduced incidence of immediate hypoxia (7.9% v 14.8%, *p* = 0.05). There was no increase in immediate hypertensive episodes (22.7% vs. 24.2%, *p* = 0.73). No safety concerns were identified.

**Conclusion:**

An attenuated fentanyl and augmented rocuronium dosing regimen showed no difference in absolute hypotensive episodes in a mixed cohort of medical and trauma patients. In trauma patients, the new regimen was associated with an increased FPS rate and reduced episodes of immediate hypoxia. Further research is required to understand the impact of such drug dosing in the most critically ill and injured subpopulation.

## Background

Airway management is a crucial component of the enhanced interventions delivered by prehospital critical care teams. The ability to provide prehospital anaesthesia (PHEA) has likely been in development since approximately the 1960s. It continues to have inherent risks and has changed significantly since then [1]. Within the United Kingdom (UK), guidelines exist to help improve safety, and there is recognition that continual audit and development are needed [2]. 

Drug dosing has remained a challenge; one in five trauma patients have been shown to have an episode of hypotension within the first ten minutes of induction of PHEA [3]. In medical patients, the statistics are worse, with over 50% of patients having an episode of hypotension recorded in one study [4]. The detrimental impact of hypotension on outcomes has been observed in a number of pathologies; in traumatic brain injury concerns have been raised for more than 30 years that any early episode of hypotension can result in increased mortality [5]. In an attempt to mitigate the risks of hypotension in trauma patients undergoing PHEA, etomidate was the traditionally used induction agent [6, 7]. Over the last decade in the UK, many prehospital critical care services have transitioned to a regime involving 3 mcg/kg fentanyl, 2 mg/kg ketamine and 1 mg/kg rocuronium (‘3:2:1’) [7]. The consensus for medical patients in PHEA remains unclear; one study showed no difference between a ketamine or midazolam based regimen in rates of hypotension for post return of spontaneous circulation patients [4]. There has also been increasing evidence that a higher dose of rocuronium (≥ 1.4 mg/kg) may improve the first pass success (FPS) rate, particularly in those patients who are most unwell and have a high shock index [8]. 

In an attempt to mitigate the risks of cardiorespiratory deterioration during PHEA, our UK-based air ambulance service formally transitioned to a standardised induction regimen consisting of 1 mcg/kg fentanyl, 2 mg/kg ketamine and 2 mg/kg rocuronium (‘1:2:2’) for both medical and trauma patients [9]. The aim of this retrospective study was to evaluate the cardiorespiratory consequences and immediate outcomes of this new attenuated fentanyl and augmented rocuronium dosing regimen.

## Materials and methods

### Operational service details

Essex and Herts Air Ambulance Trust (EHAAT) is a UK-based air ambulance that is dispatched to incidents mainly within the Essex and Hertfordshire area. It covers a semiurban dispatch area with an approximately 50:50 distribution between medical and trauma cases, operating 24 h a day, 365 days a year. A physician and critical care paramedic model is utilised and teams can perform advanced critical care interventions such as PHEA and resuscitative thoracotomy. All physicians have ongoing exposure to critically ill patients and have undertaken at least six months of anaesthesia training prior to working for the service.

The new standard operating procedure (SOP) for PHEA was introduced in August 2021 as per the paper previously described [9]. The vast majority of patients receive 1 mcg/kg fentanyl, 2 mg/kg ketamine and 2 mg/kg rocuronium (up to a maximum dose of 150 mg) [9]. Within the standard operating procedure (SOP), there is scope for adjusting doses in limited cardiovascular reserve patients (such as post return of spontaneous circulation in cardiac arrest patients) or in patients requiring higher induction doses (such as burn patients) [9]. Additionally, the use of nasal apnoeic oxygenation via nasal cannula at 15 L/minute was introduced, unless there was a contraindication, and greater importance placed on the optimisation of patients prior to PHEA [9]. Governance structures exist for reviewing all patients undergoing PHEA.

### Study methodology

A retrospective study was conducted utilising EHAAT’s electronic database, completed contemporaneously by the clinicians attending the patient. An anonymised review of the computerised HEMSbase record system (HEMSbase 2.0 Medic One Systems, London, UK) was performed for two time periods: 14th December 2019 to 31st July 2021 for patients on the traditional SOP and 1st September 2021 to 19th May 2023 following introduction of the new SOP. The dosing regimen of the traditional SOP was 3 mcg/kg fentanyl, 2 mg/kg ketamine, 1 mg rocuronium, whereas in the new SOP the regimen was 1 mcg/kg fentanyl, 2 mg/kg ketamine, and 2 mg/kg rocuronium (up to a maximum of 150 mg). There was also an option for clinicians to utilise midazolam and fentanyl regimens in medical patients in the traditional SOP. The month of August 2021 was avoided due to concern that there would be an overlap of SOPs as the transition was made.

A post hoc power analysis calculation was performed to establish the sample needed to observe a decrease of 20% in hypotension based on a previous study in hypotension in medical patients (alpha value 0.05, beta value 0.2), resulting in the need to collect a dataset of 360 in each group [4]. Data relating to sex, age, weight, reason for PHEA, patient outcome, observations and drugs administered were extracted. To be a complete data set, an initial blood pressure and pulse oximetry reading (5 min prior to the documented start time of the PHEA) were needed to allow comparison to be made to establish if hypotension, hypertension or hypoxia occurred. Drugs, including fluids, were included if they were given within 5 min of the documented start time of the PHEA and within the 10 min following this as it was deemed this may have an impact on the cardiovascular system of the patient.

The primary outcome, as per the power calculation, was the incidence of absolute hypotension at any time point within the first 10 min following the induction of PHEA. Absolute hypotension was defined as a systolic blood pressure < 90 mmHg within ten minutes of intubation or, if the initial systolic blood pressure was < 90 mmHg, then a ≥ 10% drop in systolic blood pressure within the first ten minutes [3]. Secondary cardiovascular outcomes included relative hypotension, immediate hypertension (on the first reading post-PHEA), immediate tachycardia (on the first reading post-PHEA) and the use of vasopressors or fluid resuscitation within the first 10 min (including the use of metaraminol boluses, adrenaline boluses and/or the administration of fluid boluses or blood products). Relative hypotension was defined as a 20% reduction in systolic blood pressure within the first ten minutes following intubation [7]. Immediate hypertension was defined as a new systolic blood pressure > 160 mmHg or a > 20% rise in systolic blood pressure if the starting blood pressure was ≥ 160 mmHg immediately following intubation on the first reading post-PHEA [7]. Immediate tachycardia was defined as a heart rate ≥ 120 bpm following the induction of PHEA that had increased by ≥ 20% in comparison to the initial heart rate on the first reading post-PHEA.

The respiratory outcomes were defined as an immediate hypoxic episode following PHEA induction, with saturations < 90%, or, if oxygen saturations started at 90% or below, a ≥ 10% fall relative to the starting saturations; FPS rates at intubation; the number of unsuccessful intubations (including surgical airways) and the utilisation of apnoeic nasal oxygen. Additionally, a composite safety outcome was calculated to establish whether the patient had experienced absolute hypotension within the first 10 min or relative hypotension within the first 10 min or if there had been an unsuccessful first pass at intubation or a Cormack and Lehane Grade of intubation ≥ 3 had been achieved on the view. Subanalysis was performed for trauma patients and medical patients. A comparison was also made to establish the effect of fentanyl administration due to the reduced doses used in the new SOP.

### Statistical analysis

Statistical analysis was performed in Microsoft Excel (Microsoft, Seattle, Washington, USA) and SPSS statistics (SPSS Inc., Chicago, IL, USA). A test for normality was performed for continuous data (Shapiro‒Wilk), and either an independent t test or Mann‒Whitney U test was performed as necessary. Chi square analysis or Fisher’s exact test (if 2 × 2 contingencies) was performed for categorical variables; a *p* value < 0.05 was considered to indicate statistical significance. The Strengthening the Reporting of Observational Studies in Epidemiology Checklist was followed [10]. 

### Ethics and governance

Local research policies were satisfied, and the study was registered with the EHAAT research oversight committee; ethical approval was not needed due to this being a service evaluation [11]. 

## Results

A total of 841 cases were reviewed (427 traditional SOPs and 414 new SOPs) to enable sufficient complete datasets to be extracted. Following exclusions due to incomplete data sets (most commonly due to a lack of an initial blood pressure prior to PHEA), 720 patients were analysed (360 treated under the traditional SOP and 360 treated under the new SOP). The demographics of the patients are detailed in Table 1; age and weight were normally distributed.

**Table 1:** Demographics of the Included Patients

	New SOP N = 360	Traditional SOP N = 360	*p* value
**Gender**			
Male	265	270	
Female	95	90	*0.73*
**Mean age ± SD (years)**	52.3 ± 21.6	53.0 ± 18.9	*0.62*
**Mean weight ± SD (kg)**	78.7 ± 19.4	80.1 ± 18.7	*0.34*
**Type of incident**			
Medical	196	205	
Trauma	164	155	*0.55*
**Reason for PHEA**			
Low Glasgow Coma Scale	210	176	
Airway compromise	53	90	
Clinical course	13	14	
Unmanageable/agitation	28	22	
ROSC post cardiac arrest	23	18	
Respiratory/ventilatory failure	31	38	
Humanitarian	0	1	
Other	0	1	
Not stated	2	0	0.02*
**Patient outcome**			
Aircraft carry to hospital	69	66	
Ground escort to hospital	287	292	
Ground assisted#	1	1	
Pronounced life extinct on scene	3	1	*0.78*
**First pass intubator**			
Emergency medicine physician	211	214	
Anaesthetic physician	140	121	
Critical care paramedic	6	3	
Intensive care physician	3	10	
General practitioner	0	11	
Unknown	0	1	< 0.01*
**Shock index**			
< 1.0 beats/min/mmHg	306	295	
> 1.0 beats/min/mmHg	54	65	*0.32*

*Statistically significant; SD = standard deviation; ROSC = return of spontaneous circulation; # = escorted to hospital by alternative HEM service

Table 2 demonstrates the change in ratios for drugs administered between the new and traditional SOP. Propofol was utilised in one PHEA under the new SOP (100 mg) and 5 PHEAs under the traditional SOP (60 mg, 60 mg, 100 mg, 200 mg and 150 mg).

**Table 2:** Ratio of drugs administered

	New SOP	Traditional SOP	*p* value
**Fentanyl**			
0 mcg/kg	157	111	
0.5 mcg/kg	50	19	
1 mcg/kg	109	87	
2 mcg/kg	37	50	
3 mcg/kg	7	93	< 0.01*
Median dose (mcg)	50	100	
IQR (mcg)	0-100	0-200	
**Ketamine**			
0 mg/kg	34	95	
0.5 mg/kg	22	14	
1 mg/kg	156	109	
2 mg/kg	145	133	
3 mg/kg	2	4	< 0.01*
Median dose (mg)	90	80	
Range (mg)	62.5–140	0-140	
**Rocuronium**			
0 mg/kg	0	2	
1 mg/kg	51	348	
2 mg/kg	305	8	
3 mg/kg	4	2	< 0.01*
Median dose (mg)	150	80	
Range (mg)	127.5–150	70–100	
**Midazolam**			
0 mg/kg	326	291	
0.1 mg/kg	34	69	< 0.01*
Median dose (mg)	0	0	
Range (mg)	0–0	0–0	

*Statistically significant; IQR = interquartile range

There was an overall rate of absolute hypotension in the data set of 24.1% (*n* = 174); 27.3% (*n* = 56) in the medical group and 28.9% (*n* = 116) in the trauma patient group. There was no difference between the SOPs (88 episodes of new SOP vs. 86 episodes of traditional SOP, *p* = 0.93). Table 3 demonstrates the cardiovascular complications experienced, and Table 4 demonstrates the respiratory complications experienced. When fentanyl was administrated versus when it was not there was no statistically significant change in the rates of absolute hypotension, relative hypotension, immediate hypertensive episodes or immediate tachycardia between the regimens. No episodes of awareness were reported through the governance systems for any patient.

**Table 3:** Cardiovascular outcomes

	New SOP	Traditional SOP	*p* value
**Absolute hypotension episodes**			
Overall	88/360; 24.4%	86/360; 23.9%	*0.93*
Medical patients	60/196; 30.6%	56/205; 27.3%	*0.51*
Trauma patients	28/164; 17.1%	30/155; 19.4%	*0.66*
**Composite safety outcome** ^**#**^			
Overall	179/360; 49.7%	182/360; 50.6%	*0.88*
Medical patients	110/196; 56.1%	103/205; 50.2%	*0.27*
Trauma patients	69/164; 42.1%	79/155; 51.0%	*0.12*
**Relative hypotension episodes**			
Overall	136/360; 37.8%	136/360; 37.8%	*1*
Medical patients	89/196; 45.4%	77/205; 37.6%	*0.13*
Trauma patients	47/164; 28.7%	59/155; 38.1%	*0.1*
**Immediate hypertension episode**			
Overall	82/360; 22.8%	87/360; 24.2%	*0.73*
Medical patients	41/196; 21.0%	54/205; 26.3%	*0.24*
Trauma patients	41/164; 25.0%	33/155; 21.3%	*0.51*
**Immediate tachycardic episode**			
Overall	53/360; 14.7%	43/360; 11.9%	*0.28*
Medical patients	24/196; 12.2%	21/205; 10.2%	*0.53*
Trauma patients	29/164; 17.7%	22/155; 14.2%	*0.45*
Use of vasopressors/fluid resuscitation in first 10 minutes	114/360; 31.2%	98/360; 27.2%	*0.22*

*statistically significant; ^#^Composite safety outcome compromises of any episode of absolute hypotension or relative hypotension or unsuccessful at first pass of intubation or achievement of Grade 3 or 4 laryngoscopy view

**Table 4:** Respiratory outcomes

	New SOP	Traditional SOP	*p* value
**Immediate hypoxic episodes**			
Overall	31/360; 8.6%	44/360; 12.2%	*0.09*
Medical	18/196; 9.2%	21/205; 10.2%	*0.74*
Trauma	13/164; 7.9%	23/155; 14.8%	0.05*
If shock index > 1	4/55; 7%	8/66; 12.1%	*0.54*
**First pass success rates**			
Overall	332/360; 92.2%	321/360; 89.2%	*0.2*
Medical	176/196; 89.8%	187/205; 91.2%	*0.73*
Trauma	156/164; 95.1%	134/155; 86.4%	0.01*
**Unsuccessful intubations**			
Overall	2/360; 0.6%	3/360; 0.8%	*1*
Supraglottic airway utilised	0/360; 0%	3/360; 0.8%	*0.25*
Surgical airway performed	2/360; 0.6%	0/360; 0%	*0.5*
**Apnoeic nasal oxygen utilised**	280/360; 77.8%	180/360; 50%	< 0.01*

*statistically significant

## Discussion

This study has shown that protocolised dosing adjustment as part of a PHEA SOP modification, with attenuated fentanyl and augmented rocuronium dosing, was not associated with fewer absolute hypotensive episodes across a mixed medical and trauma cohort. However, there was a statistically significant decrease in immediate hypoxic episodes and an increased FPS rate in trauma patients. Importantly, there was no increase in immediate hypertensive or immediate tachycardic episodes with attenuated fentanyl dosing. The move to ubiquitous use of ketamine in both medical and trauma patients as part of the new SOP was also shown to have comparable cardiovascular profiles and rates of adverse events. Whilst this study was performed in the pre-hospital setting it is likely these findings would be replicated within an in-hospital setting.

The overall rate of absolute hypotension is not dissimilar to a previous trauma study in a similar population group (28.9% in trauma group versus 21.8% previously published) but is less than a previously published medical study (27.3% medical within 10 min of PHEA vs. 50% published previously within 30 min of PHEA) [3, 4]. Even within the new SOP group, there remains a 24.4% absolute hypotension rate, and efforts must continue to improve this with the known poorer effect on patient outcomes with every episode of hypotension [5, 12]. There is as yet no significant difference between the use of resuscitative techniques around the PHEA, and we would encourage clinicians to continue to consider early optimisation of their patients prior to anaesthesia. Clinicians must be aware that conversion to positive pressure ventilation can result in hypotension, particularly if a patient is underfilled. The fear of vasopressors in trauma remains ever-present, but there is beginning to be some evidence in support of them, and it is now recommended with caution in trauma by the European guidelines on the management of major bleeding and coagulopathy [13–15]. Optimisation of a patient will depend on the underlying pathology and can include vasopressors, blood products and fluids. However, whatever methods are utilised by clinicians, we will continue to encourage the avoidance of any hypotension in order to improve outcomes in our patients, particularly those with a traumatic brain injury [5]. 

It is also important that there has been no change in hypertensive or tachycardic episodes with the change in SOP. The rates of hypertension seen are higher than in a recent study in trauma patients, which showed a rate of 11.9%, although the definition of hypertension notably differed as hypertension was defined as a systolic blood pressure > 160mmHg in the Sagi et al. (2023) study versus 180mmHg in this study [16]. The evidence of harm for hypertension, particularly in traumatic brain injury, is limited, and there remains a lack of consensus on whether it requires treatment or whether it is simply a mechanism by which the brain maintains cerebral perfusion pressure and is an area that requires additional evaluation [17]. In the post return of spontaneous circulation patients, there may be concern that tachycardia will put strain on the heart’s oxygen demand, although a recent study has shown improved outcomes in those with an increased heart rate [18]. Hypertension and tachycardia can both be consequences of laryngoscopy and while this study was not powered explicitly for these outcomes, it is a useful finding that can be further investigated in the future. Indeed, it may be that fentanyl, traditionally added to attenuate the laryngoscopy response, may not be required at all, or for certain patient populations such as burns. It is recognised that the time given to fentanyl to work in a rapid sequence induction is unlikely to be sufficient; indeed a recent study in elective surgical patients showed that ketamine was an alternative to fentanyl alongside propofol for attenuating this response and it resulted in less hypotension [19]. 

The improvement seen within trauma patients at the FPS rate and decreased incidence of immediate hypoxia is noteworthy. The FPS rate with the new SOP for trauma (95.1%) is still lower than that in previous studies, but this may be influenced by the changing pathologies seen with the UK trauma scene, as the major trauma network has developed over the past 10 years [7]. A dose of 1.0 mg/kg rocuronium is technically underdosing, and the UK Difficult Airway Society encourages adequate neuromuscular blockade to optimise intubating conditions [20]. The increased dosing regimen in the new SOP aligns with the improved FPS rate findings seen in emergency department intubations [8]. It also correlates with a recent paper from the Greater Sydney Area Helicopter Emergency Medical Service, who introduced a higher dose of rocuronium at the end of 2017, and showed there was a trend to fewer inadequately paralysed patients with a higher dose of rocuronium [21]. Hypoxia can also have an important influence on outcomes, particularly in traumatic brain injury mortality, and we would therefore encourage the use of a higher dose of rocuronium in patients requiring an emergency anaesthetic [5]. In addition, it is encouraging to see the improved use of nasal cannula oxygen during the apnoeic period to try and prevent desaturations with the new SOP [9]. Apnoeic oxygenation for the emergency anaesthesia of prehospital trauma patients has been previously shown to reduce the frequency of per-intubation hypoxia for patients starting with normal oxygen saturations and also to have a significant benefit for patients in the recovery phase of anaesthesia who were severely hypoxic prior to intubation [22]. Overall, the findings would suggest that while these are small changes if there is the possibility of preventing morbidity in our patients, they are to be encouraged as standard practice.

The lack of effect of statistically significant difference in immediate hypoxic episodes between the SOPs when the shock index was > 1.0 beats/min/mmHg is perhaps a surprise. The largest effect of increased rocuronium dosing improvement was in patients who were initially hypotensive [8]. A shock index greater than 1.0 beats/min/mmHg is known to be associated with increased hypotensive episodes, and we may therefore have expected an improvement in this group with the new dosing regimen [4]. However, it may be that the study is underpowered to establish this difference, and this could be an area for further research.

### Limitations

This is a retrospective study and relies on the accuracy of the data recorded. This is potentially prone to some recording bias. Observations are directly uploaded from the monitor used within the service, so it is hoped that mistakes with these are limited, but exact timings of drug administration may not be fairly represented. It is also recognised that the use of non-invasive blood pressure can result in difficulties, for example, depending on the size of the patient and if there is movement. It is hoped that with the number of cases included in the study, any discrepancies are equalised over both SOPs and therefore have a limited impact on results, but it is appreciated that different pathology patterns are amongst some of the factors that may have influenced results. Similarly, whilst there was a statistically significant difference in the reasons for intubation, it is likely that this actual difference is minimal; clinicians often use airway compromise and a low Glasgow Coma Scale interchangeably as a reason for intubation, as a low Glasgow Coma Scale will in itself result in airway compromise. The difference relating to the base speciality of the intubators, whilst statistically significant, is also likely to be minimal in regards to the clinical impact; all of the clinical staff employed by EHAAT must meet a minimal anaesthetic standard and undergo rigorous training and governance, as suggested by a 2017 meta-analysis [23]. Whilst we therefore appreciate both factors could be influential, particuarly in regards to FPS rates and immediate hypoxia, it is something that will require ongoing investigation.

A total of 121 cases were excluded due to incomplete data sets, which could impact some of the study findings. For the new SOP, 38 medical patients were excluded (out of 54 patients excluded), and the remainder were either accidental injuries or following a road traffic collision. For the traditional SOP, 42 medical patients were excluded (out of 65 patients excluded), again with the remainder mostly being either accidental injury or road traffic collision. The concern is that the most unwell patients, with likely the highest shock index, have been excluded due to incomplete data sets and therefore could dilute the benefit of the new SOP. This concern is supported by previous work involving hypovolaemic trauma patients which was also limited by incomplete data sets [24]. Crewdson et al. (2018) found only 45.5% of patients undergoing PHEA had a documented numerical value for initial systolic blood pressure versus 85.6% in this study [24]. The PHEA SOP was modified with the expectation that it would likely benefit most such critically ill and injured patients with significant pathophysiological derangement, and it is recognised that the conclusions drawn may be limited as a result. Any future research that can establish if this is the case is of great importance as, anecdotally, it is the patients with no recorded initial blood pressure that clinicians are most cautious about proceeding to PHEA with. The trends seen in the trauma patients are encouraging, but it is hoped that these findings may be replicated across all patient groups. Several UK HEM services utilise invasive blood pressure monitoring, and such continuous blood pressure monitoring during PHEA may be of benefit to patients and subject to future study [25]. Alongside this it is recognised that whilst an SOP exists there is always an element of clinician discretion in the dosing of patients, resulting in the variation seen in drug regimens. It would be wrong to remove this discretion and, as such, this is a real-life working of an SOP.

Finally, it is important to note that a change to both the dose of fentanyl and rocuronium has been made at the same time. It is therefore difficult to elicit which agent has resulted in the changes observed, or indeed if it is the combination of them both. It may be assumed that the fentanyl change has the greatest effect on the cardiovascular system and the rocuronium on the first pass success, but these are assumptions only. Moreover, the widespread use of apnoeic oxygenation has also been introduced, and its exact effect is unclear.

## Conclusion

SOP modification and transition to a standardised 1 mcg/kg fentanyl, 2 mg/kg ketamine and 2 mg/kg rocuronium (‘1:2:2’) regime, from a historical ‘3:2:1’ regimen, was not associated with a reduced incidence of haemodynamic adverse events during PHEA in a mixed cohort. In the trauma population, the ‘1:2:2’ regime was associated with an increased FPS rate and reduced episodes of desaturation. No safety concerns were identified across a wide range of scrutinised variables. Further research is needed to capture the potential benefit to the most critically unwell and injured patients, and the use of continuous invasive blood pressure monitoring may aid this. It should also be remembered that the process of PHEA is wider than the drug regimen alone and should include optimisation of the patient across a number of additional parameters. It is likely that this attenuated fentanyl and augmented rocuronium regimen is useful for both pre-hospital and additional work is required to establish any potential benefit in-hospital patients.

## Data Availability

The datasets generated during the current study is not currently available.
